# The Preforming of an Interlaminar Toughened Carbon Fiber/Bismaleimide Resin Composite by a Benzoxazine-Based Tackifier

**DOI:** 10.3390/ma15124196

**Published:** 2022-06-13

**Authors:** Yaxian Zi, Yulian Zhang, Weidong Li, Gang Liu, Yujing Zhou, Hua Bai, Xiaolan Hu

**Affiliations:** 1 College of Materials, Xiamen University, Xiamen 361005, China; a865749706@163.com (Y.Z.); 20720191150109@stu.xmu.edu.cn (Y.Z.); baihua@xum.edu.cn (H.B.); 2 Key Laboratory of Advanced Composite, Composite Technology Center, AVIC Composite Corporation Ltd., Beijing 101300, China; liwdhappy@163.com (W.L.); liugang@iccas.ac.cn (G.L.); 3 Center for Advanced Low-Dimension Materials, Donghua University, Shanghai 201620, China; 4 State Key Laboratory of Advanced Forming Technology and Equipment, Beijing National Innovation Institute of Lightweight Ltd., Beijing 100083, China; zhouyujingcam@126.com; 5 DE Zhou Branch of Beijing National Innovation Institute of Lightweight Co., Ltd., Dezhou 253049, China

**Keywords:** benzoxazine, tackifier, preforming, toughening, composites

## Abstract

When thermoplastic resin-toughened carbon fiber (CF) composites are formed by liquid resin transfer molding (RTM), the conventional methods cannot be used to set the fabric preform, which affects the overall mechanical properties of the composites. To address this challenge, the benzoxazine-based tackifier BT5501A was designed, a preforming–toughening bifunctional CF fabric was fabricated by employing thermoplastic polyaryletherketone (PEK-C), and an aviation RTM-grade bismaleimide (BMI) resin was used as the matrix to study the effect of the benzoxazine-based tackifier on the thermal curing property and heat resistance of the resin matrix. Furthermore, the preforming and toughening effects on the bifunctional CF fabric reinforced the BMI resin composites. The tackifier BT5501A has good process operability. The application of this tackifier can advance the thermal curing temperature of the BMI resin matrix and decrease the glass transition temperature of the resin, compared to that of the pure BMI resin. Furthermore, when the tackifier was added into the CF/PEK-C/BMI composites, the obtained CF/BT5501A/PEK-C/BMI composites had comparable compression strength after impact, pit depth, and damage area, compared to the CF/PEK-C/BMI composites, while the tackifier endowed the fabric preform with an excellent preforming effect.

## 1. Introduction

Toughening is always an important issue in high-performance thermoset resin matrix composites. In situ toughening through thermoplastic resins, rubbers, nanomaterials, etc., can effectively improve the overall toughness of composites. However, this in situ toughening method will greatly increase the viscosity of the composite resin matrix, which is not suitable for the resin transfer molding (RTM) process [1]. As a cost-effective manufacturing technology for advanced composite materials, liquid molding technology represented by RTM has become the mainstream of research and development in the composite materials field [2]. Since the RTM process requires a special resin with ultra-low viscosity, the widely used RTM resins are generally not toughened due to process problems. Actually, the interlaminar toughening method is a good strategy to solve the contradiction between the low viscosity of RTM resin and the high toughness of composite materials [3,4,5,6,7]. Thermoplastic resin films, powder, and thermoplastic resin nano-nonwoven fabrics can toughen the composites well and have been successfully applied to multiple aerospace composite parts [8,9,10,11].

The RTM process first requires preparing a fiber preform, of which the shape is similar to the composite part; then, an infiltration of the fiber preform and resin is accomplished by the rapid flow of the pressurized resin in a closed mold cavity. However, when the thermoplastic resin film or powder is placed between the layers of the fabric for interlaminar toughening, due to the lack of surficial adhesion, the fiber preform cannot be tackified well. This causes the fibers in the fabric to become prone to buckling and dislocating when the fiber preform is injected with resin under high pressure, and eventually affects the final physical properties of the composite parts. Such a case is more prominent for large-size composite parts. To ensure a good predetermined shape of the fiber preform, and subsequently, a high-quality composite part, a tackifier is required.

The tackifier should not only have a good preforming effect to ensure mold loading, but also good process operability and minimal impact on the physical properties of the resin matrix. There are both solution-type or a dry powder-type tackifiers. Solution-type tackifiers will penetrate easily into the fabric to cause difficulty in resin flow; in contrast, dry powder-type tackifiers adhere to the fabric surface to set the preform. Therefore, the dry powder-type tackifier is more suitable for the RTM process.

Our previous research revealed that the thermoplastic polyaryletherketone (PEK-C) resin has an obvious modification effect on the toughness of the carbon fiber/bismaleimide (CF/BMI) composites prepared by RTM [4]. In order to match the high heat resistance and mechanical properties of the bismaleimide resin, we designed a benzoxazine-based tackifier with high heat resistance to improve the preforming property of the RTM preforms. An aviation RTM-grade bismaleimide resin was selected as the matrix for fabricating the interlaminar toughened composites. The preforming effect of the tackifier on the carbon fiber preform toughened with the thermoplastic PEK-C resin powder was studied, and the impact of the tackifier on the thermal curing and heat resistance properties of the bismaleimide matrix was investigated as well. Meanwhile, the overall toughness of the thermoplastic resin-toughened CF/PEK-C/BMI composites with the tackifier were evaluated.

## 2. Materials and Methods

Benzoxazine (BOZ) resin was supplied by the College of Polymer Science and Engineering, Sichuan University. Bismaleimide (BMI 6421) resin was supplied by the Beijing Institute of Aeronautical Materials. U3160 unidirectional non-woven carbon fiber (CF) fabric was supplied by Weihai Tuozhan Fiber Co., Ltd. Thermoplastic phenolphthalein polyaryletherketone (PEK-C) resin was supplied by Jilin University. The Benzoxazine-based tackifier BT5501A was homemade.

The curing process for BMI resin and BMI–BT5501A resin is 150 °C/1 h + 160 °C/1 h + 180 °C/2 h + 200 °C/8 h. The dosage of the tackifier is 10% (mass ratio) of the BMI resin. The PEK-C is a toughening agent, and the BOZ resin is an auxiliary binder. The two components were dissolved in tetrahydrofuran (THF) to obtain a mixed solution. The mixed solution was sprayed onto the surface of the CF fabric using a spray gun, and air-dried at room temperature to obtain a fabric with a single-sided adhesion of the toughening agent. The tackifier BT5501A powder was prepared according to the literature [12]. The BT5501A powder was evenly spread mechanically on the other side of the CF fabric without the toughening agent; then, the fabric was baked to obtain a bifunctional CF fabric with a toughening agent attached to one side and a tackifier attached to the other side.

The softening point of the tackifier is determined referring to the GB/T12007.6-1989 method for epoxy resin, using a FY-28A intelligent asphalt softening point tester. The curing exothermic process of the sample was examined using the DSC Q10 (Thermal Analysis & Rheology Instruments Inc., New Castle, DE, USA) under the conditions of a N_2_ atmosphere and a heating rate of 5 °C/min. The dynamic thermomechanical analysis was performed using the American TA DMA Q800 at a frequency of 1 Hz, a heating rate of 5 °C/min, and under a double cantilever mode. The preforming effect was evaluated in accordance with the literature [13,14]. Ultrasonic C-scanning of composite laminates was performed using an ultrasonic C-scanner from KK, Germany. The compression after impact (CAI) strength test referred to the composite test standard ASTM D7137/D7137-05, the sample size was 150 mm × 100 mm, and the lay-up configuration was (45°/0°/−45°/90°)_3S_; the sample was impacted by a steel punch having a diameter of 16 mm, and the impact energy was 6.67 J/mm. The microstructure of the sample was observed by a Quanta 600 environmental scanning electron microscope from FEI, the Netherlands. The sample was immersed in THF for 72 h, dried, and then sprayed with gold for observation.

## 3. Results

### 3.1. Design and Properties of Benzoxazine-Based BT5501A Tackifier

The design of the tackifier is based on the performance of the resin matrix and the preforming requirements of the fiber preform. Usually, in order to achieve good presetting of the fiber preform, in the preparation of carbon fiber-reinforced resin matrix composites, the resin matrix itself—or a modified resin with similar heat resistance properties—is selected as the tackifying agent for the fiber preform, resulting in good compatibility between the tackifier and the resin matrix, and in little impact on the heat resistance of the composites. Due to the low viscosity requirements of RTM resin, BMI resin cannot be toughened through in situ methods. Therefore, structural toughening methods such as interlayer toughened CF/BMI composites by thermoplastic resin turned out to be very important. Owing to the poor adhesion at both room temperature and low heating temperatures, the BMI resin itself cannot tackify the CF fabric. Considering the characteristics of the BMI resin matrix composites, as well as the technical requirements of the RTM process, it is very helpful to design a tackifier that can meet the requirements of high temperature resistance, and at the same time meet the toughening and preforming requirements in the RTM process.

From the analysis of the molecular structure, resins with high heat resistance usually have low room-temperature adhesion, and consequently, a poor preforming effect on the CF fabric. In the high-performance thermosetting resin matrix, although the epoxy resin has good adhesion at room temperature, its heat resistance is much lower than BMI resin. In addition, the amount of the tackifier is usually about 10% (mass ratio) of the resin matrix, which easily causes an obvious decrease on the heat resistance of the composites. In contrast, the benzoxazine (BOZ) resin has a high heat resistance, a mechanical strength at high temperature (180 °C) close to that of the BMI resin, no release of small molecular substances during the ring-opening polymerization, as well as approximately zero heat-shrinkage of the cured product and excellent processability—almost the same as epoxy resin [15,16,17]. On account of these advantages, we have designed a BOZ-based tackifier named BT5501A using diphenylmethanediamine benzoxazine resin as the matrix.

**Figure 1:**
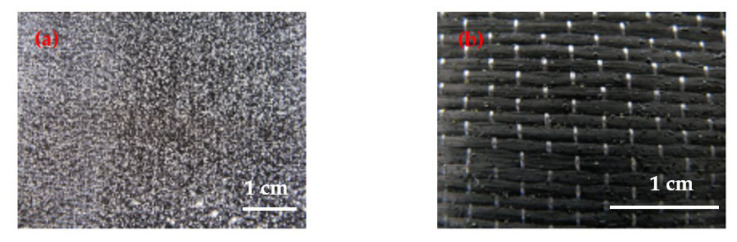
Images of carbon fiber fabric surfaces with benzoxazine-based tackifier BT5501A, (**a**) before baking and (**b**) after baking.

The operability of the BT5501A tackifier was examined for the RTM process. The softening point temperature of BT5501A is 74.2 ± 0.5 °C. It was pulverized at room temperature, sieved, and evenly spread on the surface of the CF fabric, as shown in Figure 1a. The fabric was then baked near the softening point, and after baking, the tackifier turned into microspheres adhered to the surface of the fabric, as shown in Figure 1b. These microspheres cannot be easily detached from the surface of the fabrics. The results demonstrate the suitable operability of the tackifier.

### 3.2. Effects of Tackifier on the Thermal Property of BMI Resin Matrix

BMI resin is one of the most important resin matrices in high-performance aeronautical composites. In addition to its excellent mechanical properties and molding process properties, BMI resin also has excellent heat resistance. Because about 10% of the setting agent is used in the composite resin, it may have a significant negative impact on the heat resistance of the BMI resin matrix. To balance the adhesion of the tackifier and the heat resistance of the BMI–BT5501A-modified resin, the effects of the tackifier on the thermal curing and dynamic thermomechanical properties of the BMI resin matrix were investigated.

**Figure 2:**
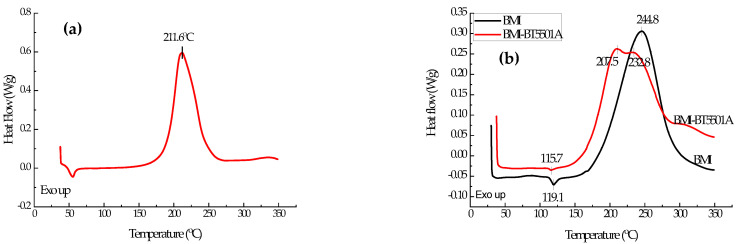
DSC curves of the benzoxazine-based BT5501A tackifier (**a**), bismaleimide, and bismaleimide–BT5501A composite resin (**b**).

Figure 2a features a DSC curve of the benzoxazine-based BT5501A tackifier showing a maximum exothermic peak at 211.6 °C. The curing of the diamine benzoxazine resin involves a ring-opening reaction of the oxazine ring, forming a structure containing a carbon cation and a phenolic hydroxyl group. The chain propagation is achieved by the electrophilic substitution of carbon cations, and the active hydrogen on the phenolic hydroxyl group promotes the further opening of the other benzoxazine rings, leading to an electrophilic self-polymerization and eventually producing polybenzoxazine [18]. The exothermic peak at 211.6 °C in Figure 2a should represent the ring-opening solidification process of the tackifier.

The black curve in Figure 2b is the DSC curve for BMI with a maximum exothermic peak at 244.8 °C and a small endothermic peak at 119.1 °C. The BMI resin used herein is an allyl bisphenol A-modified diaminodiphenylmethane-type bismaleimide resin. For preparing the resin, the allyl bisphenol A and the diaminodiphenylmethane-type bismaleimide powder were prepolymerized at 130 °C/15 min. Thus, the small endothermic peak at 119.1 °C should be ascribed to the dissolution of unpolymerized bismaleimide powder into an allyl bisphenol A solution. Without a catalyst, the copolymerization of bismaleimide and allyl bisphenol A is mainly the addition reaction between the C=C bond and a terminal alkenyl group, and the reaction temperature is around 150–200 °C [19]. The Diels–Alder reaction of the C=C bond between allyl and maleimide rings contains two steps. The first step is a diene addition reaction of a maleimide group and an allyl group, forming a 1:1 addition product. The reaction generally occurs at about 120 °C, with a low activation energy. The second step is a Diels–Alder addition reaction of the BMI with the addition product to form a ladder network structure; the reaction temperature is higher than 200 °C. These characteristics require that the post-treatment temperature of the allyl compound-modified BMI resin should be higher than 230 °C [20].

It is worth mentioning that the allyl bisphenol A alone is difficult to polymerize, even with different initiators. It was difficult to gel when treated at 200 °C for several hours. In addition, a DSC analysis of allyl bisphenol A showed no reaction exotherm peak until 280 °C [21].

**Figure 3:**
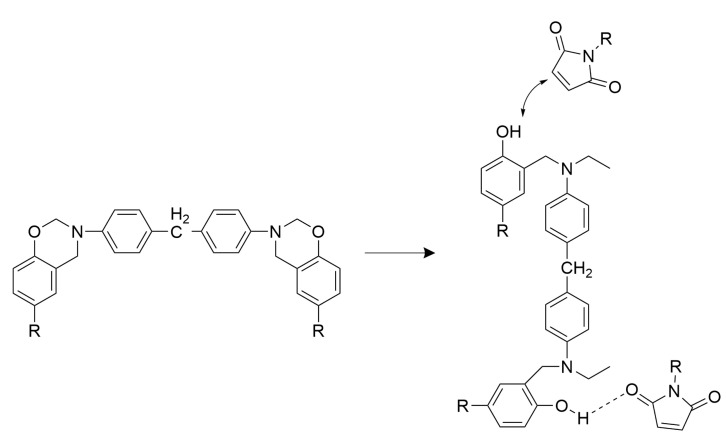
Possible chemical reactions between benzoxazine and bismaleimide resin [22].

**Figure 4:**
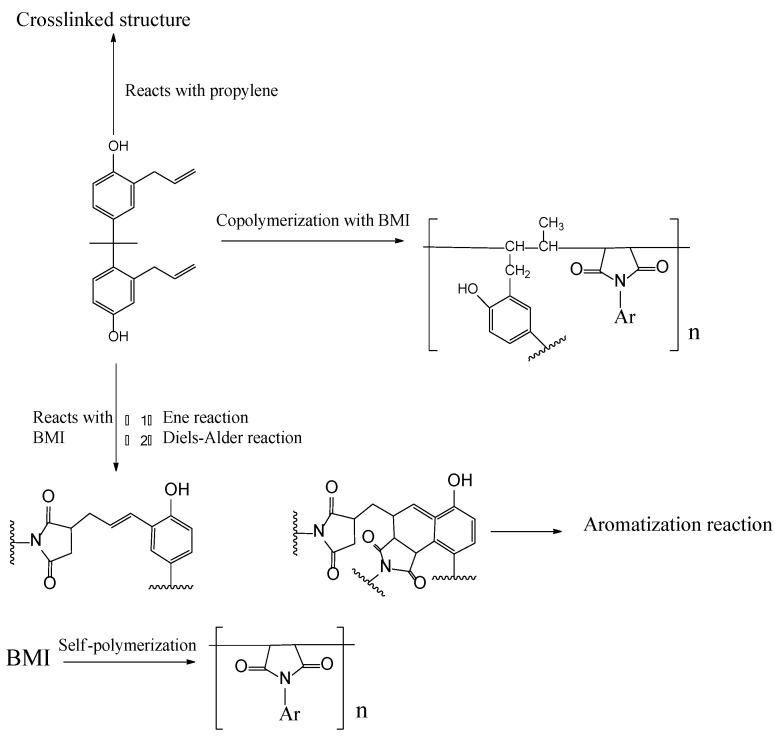
The reactions of allyl bisphenol A-modified bismaleimide resin [25].

The main exothermic peak of the curing reaction of BMI–BT5501A appeared earlier than that of BMI resin, as shown in Figure 2b, including two main peaks at 207.5 °C and 232.8 °C, respectively. The reaction between the BMI resin and the BOZ is complicated; the hydroxyl group formed by the ring-opening of the benzoxazine not only reacts with the double bond on the maleimide, but also forms a hydrogen bond with C=O on the maleimide, as shown in Figure 3 [22]. The effect of BMI on the BOZ curing is related to the interaction between them. According to our analysis, the phenolic hydroxyl group may play a key role. The phenolic hydroxyl group is mainly derived from allyl bisphenol A and BOZ after ring opening. The phenol can promote the ring-opening of BOZ [23]; therefore, the solidification exothermic peak of BOZ is slightly advanced—to 207.5 °C. Meanwhile, the oxygen anion and imine ion formed by the ring-opening of BOZ catalyze the polymerization of BMI, resulting in a lower curing temperature for the resin, wherein the oxygen anion plays a major catalytic role [24]. This causes the BMI-cured exotherm to move forward to 232.8 °C. The endothermic peak at 115.7 °C should be ascribed to the dissolution of unpolymerized BMI powder into allyl bisphenol A solution. The exothermic broad peak above 300 °C may be the self-polymerization of the excess allyl compound. Generally, the allyl compound is relatively stable, and the self-polymerization reaction occurs at 300–350 °C, as shown in Figure 4 [25]. In addition, it is also reported that the different ratios of BOZ and BMI resin can also lead to different reactions [22].

**Figure 5:**
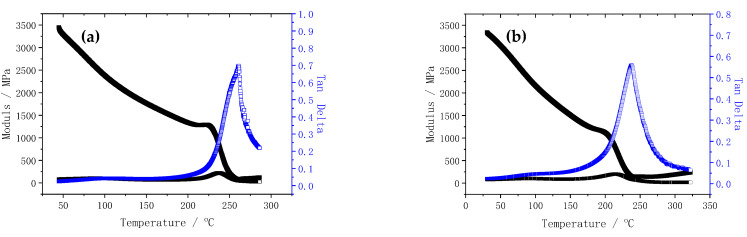
Dynamic thermomechanical analysis (DMTA) of BMI (**a**) and BMI–BT5501A (**b**).

Figure 5 shows the dynamic thermomechanical (DMTA) curves of the BMI resin and the BMI–BT5501A composite resin. The glass transition temperature (T_g_) of BMI is 259.2 °C, while the T_g_ of the BMI–BT5501A composite resin is 237.8 °C, which is 21.4 °C lower than that of the BMI resin matrix. To achieve a good preforming effect, the amount of tackifier is usually up to 10% by weight, based on the quantity of the resin matrix. The reduction in T_g_ of the BMI–BT5501A composite resin may have a negative impact on the long-term working temperature of the composites. BMI resin and high-temperature epoxy resin are the most important heat-resistant resin matrices for aerospace composites. Because of the good adhesion of epoxy resin, the bulk resin is usually used directly as a tackifier for the fiber preform. Therefore, the heat resistance of the epoxy-based composite material is generally not affected by the usage of a tackifier. In contrast, the BMI resin has poor adhesion. The addition of a tackifier achieves a good preforming of the fiber preform; at the same time, it causes a certain loss in the heat resistance of the BMI resin. To balance this problem, the heat resistance of the composite material may be compensated for by increasing the degree of post-curing of the composite resin system.

### 3.3. Preforming Effect of Tackifier on CF Fabric

**Figure 6:**
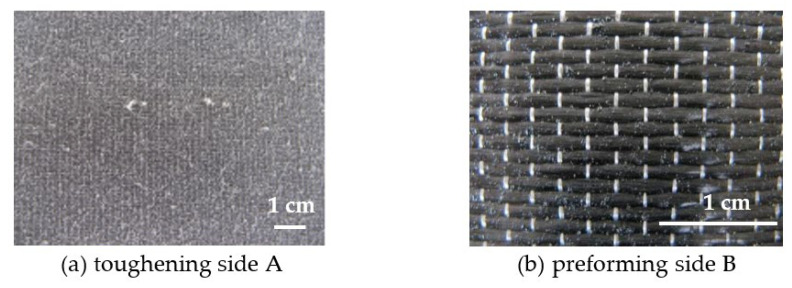
Images of the bifunctional CF fabric surface with toughening agents (**a**) and preforming agents (**b**).

When a thermoplastic resin is used to toughen the RTM composites, neither the thermoplastic resin film nor the powder-toughened CF preform can be conventionally preformed. To achieve both interlaminar toughening and preforming, we have designed a preforming–toughening bifunctional CF fabric, as shown in Figure 6. The CF fabric was obtained by dissolving the toughening component PEK-C and the auxiliary binder BOZ in THF, and depositing on one side of the fabric by atomization spraying. On the other side of the fabric, the tackifier BT5501A was sprayed and baked to form small particles, with particle sizes between 200–600 um, obtaining a bifunctional CF fabric having a toughening function on the A side and a preforming function on the B side.

The surface morphology of the A side of the fabric is shown in Figure 6. The toughening agent was uniformly dispersed on the surface of the fibers, and the fiber tows were still loose to maintain good resin wettability. The flexibility of the fabric was reduced after loading with the toughening agent, but still sufficient to ensure good operability. The morphology of the B side is shown in Figure 1b and Figure 6b. After baking, the surfaces of the carbon fibers were attached by tackifier microspheres, which is beneficial to the bonding and preforming of the fabric.

**Figure 7:**
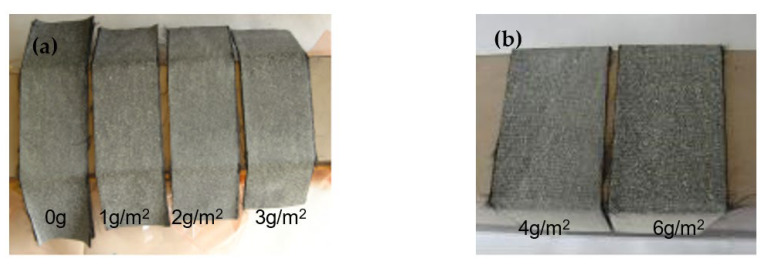
Images of (**a**,**b**) the bifunctional CF fabrics treated at 80℃/45 min in vacuum for the preforming evaluation.

The preforming effect of this bifunctional CF fabric was then examined. The fibers were laid up according to the method (45°/0°/−45°/90°)_3S_. The preforming effect of the tackifier was evaluated by its spring-back angle. As shown in Table 1, the spring-back angle decreased significantly, from 43.7° to 34.5°, when the content of BT5501A was just 1 g/m^2^ of the fabric, indicating a certain preforming effect compared with the original fabric without a tackifier. The spring-back angle of the fabric gradually decreased as the amount of the tackifier increased, and the fabric layup maintained good integrity. When the amount of the tackifier increased to 4 g/m^2^ or more, the fabric was still tightly adhered to the mold after 48 h, as shown in Figure 7. Although the flexibility of the fabric was reduced with a toughening agent, the preforming effect was excellent after using the BT5501A tackifier.

### 3.4. Toughness Evaluation of Preforming–Toughening Bifunctional CF/PEK-C/BMI Composites

**Figure 8:**
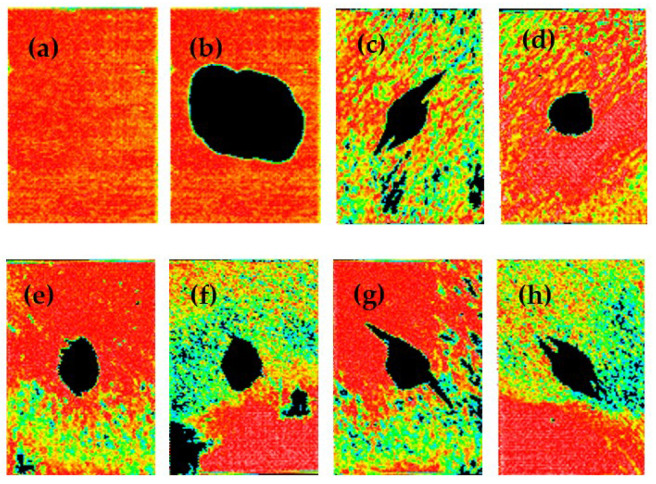
C-scanning images of (**a**,**b**) CF/BMI composites, (**c**,**d**) CF/PEK-C/BMI, (**e**–**h**) CF/BT5501A/PEK-C/BMI.

It is recognized in the literature and in industry that compression after impact (CAI) is an index that can comprehensively evaluate the overall toughness of composite materials. The toughening effect of the bifunctional fabric has been evaluated by compression after impact strength testing. Figure 8 and Table 2 are the CAI test results for the composites prepared by the bifunctional fabric and BMI resin. As shown in Figure 8, the CF/BMI composite laminate shows good internal quality, because of no doping of the CF/BMI fiber preform. When the composites were prepared by the RTM process, the injection could be performed smoothly, resulting in a good quality of composite laminate. However, after impact, the internal damage of this CF/BMI composite laminate was serious, resulting a large damage area, as shown in Figure 8b. The impact pit depth of the CF/BMI composite laminate was 0.9 mm, as shown in Table 2.

The CF/PEK-C/BMI composite laminates were prepared by adding PEK-C powder as the interlaminar toughening component. Since PEK-C was used between each layer of fabric, the actual layer thickness of the fiber preform was increased. To maintain the in-plane mechanical properties of the composites, it was necessary to maintain the thickness of the composite laminate. The fabric inside the preform became tighter, which affected the injection and flow of the resin, resulting in a slightly worse internal quality of the CF/PEK-C/BMI composite laminate compared to the CF/BMI. The experimental results show that the in-plane mechanical properties of such composite laminates have not been significantly reduced [4]. However, the CAI strength of the CF/PEK-C/BMI composite laminate was significantly improved by the interlaminar toughening of the thermoplastic resin, and the damage area of the composites after impact was also significantly decreased—by more than that of the CF/BMI composite laminate, as shown in Figure 8c,d. Related studies in our research group have also shown that the PEK-C can achieve interlaminar toughening of BMI resin by forming a two-phase structure with the BMI resin matrix [4,26].

**Figure 9:**
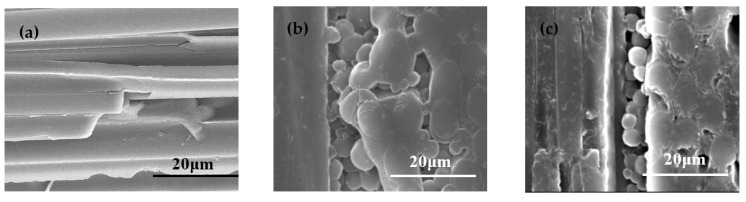
SEM images of CF/BMI (**a**), CF/PEK-C/BMI (**b**), and CF/BT5501A/PEK-C/BMI (**c**) composites.

Furthermore, the CF/BT5501A/PEK-C/BMI composite laminates were prepared through the RTM process, using the bifunctional CF fabric and BMI resin. The toughening effect was compared with CF/PEK-C/BMI composites and CF/BMI composites. The results show that the composite laminates prepared by the bifunctional fabric can achieve an excellent preforming effect, with good injection properties for the fiber preform. Moreover, from the results of Figure 9e–h, the quality of the CF/BT5501A/PEK-C/BMI composite laminates was comparable to that of the CF/PEK-C/BMI composite laminates, and the damage area of the composites was also close. Both damage areas were significantly reduced compared to that of the CF/BMI composite laminates.

From the results in Table 2, the CAI strength of the CF/BMI composite laminate without toughening was 157.5 MPa, the CAI strength of the CF/PEK-C/BMI composite laminate was significantly increased to 217.7 MPa, and the CAI strength of the CF/BT5501A/PEK-C/BMI composite laminate also reached 211.2 MPa, which was close to the CF/PEK-C/BMI composite laminate. The impact pit depth of the CF/BMI composite laminate was 0.90 mm, and was 0.61 mm for the CF/PEK-C/BMI composite laminate. The impact pit depth of the CF/BT5501A/PEK-C/BMI composite laminate was 0.69 mm, which is also close to the CF/PEK-C/BMI composite laminate, and much smaller than CF/BMI composite laminate. We can conclude that the CAI strength and impact pit depth of the CF/BT5501A/PEK-C/BMI composite laminate are comparable to those of the CF/PEK-C/BMI composite laminate, but much better than those of the CF/BMI composite laminate. The CF/BT5501A/PEK-C/BMI composite laminate not only shows a very good preforming effect, but also achieves a, interlaminar toughening effect comparable to the CF/PEK-C/BMI composites.

The internal micro-structures of the composite laminates were studied by immersing and etching the samples in tetrahydrofuran (THF) for 72 h. As shown in Figure 9, for the CF/PEK-C/BMI composites, the thermoplastic resin PEK-C in the interlayer region of the CF fabric was dissolved by THF, leaving a thermosetting BMI cross-linked structure. As stated in our previous work [4,27], the result indicates that in the interlaminar region of the fabric, which is thermoplastic resin-rich, the bicontinuous phase structure is the PEK-C component, and the spherical phase structure is the BMI component, as shown in Figure 9b. In the 0° ply close to the interlayer, phase separation occurs, but in the deeper side of the ply, the matrix remains the BMI component in the continuous phase, indicating that the bicontinuous microstructure mainly exists in the interlaminar regions of the fabric. Such CF/PEK-C/BMI composites can form a two-phase structure in the inter-layer, with BMI as the dispersed phase and PEK-C as the continuous phase, achieving interlaminar toughening [26,28].

From the image of the internal microstructure of the CF/BT5501A/PEK-C/BMI composite laminate in Figure 9c, it is also found that the thermosetting BMI–BOZ was dispersed in the composites, and the thermoplastic PEK-C was the continuous phase, resulting in a two-phase structure. The result indicates that although the composite laminate was added with ~10% by weight of the tackifier in the BMI resin matrix, the interior of the composites remained in a similar structure as in the CF/PEK-C/BMI composites. It is this two-phase structure in the thermoplastic resin-rich interlaminar region that leads to the interlaminar toughening of composite laminates.

## 4. Conclusions

The powder-type benzoxazine-based tackifier BT5501A demonstrates good operability for the RTM process. It has a suitable softening temperature, and forms small microspheres adhering to the surface of the carbon fiber fabric after baking. The application of the BT5501A tackifier can advance the curing reaction peak of bismaleimide resin, while decrease the glass transition temperature of bismaleimide resin from 259.2 °C to 237.8 °C.

The tackifier shows an excellent preforming effect on the fiber preform prepared by the preforming–toughening bifunctional carbon fiber fabric. Both of the CF/BT5501A/PEK-C/BMI composites and the conventional thermoplastic resin interlaminar toughened CF/PEK-C/BMI composites are more significantly toughened than the un-toughened CF/BMI composites; the compression after impact (CAI) strengths of the composites all increased by more than 30%. Moreover, the CF/BT5501A/PEK-C/BMI composites and the CF/PEK-C/BMI composites have comparable CAI strengths, pit depths, and damage areas. These results demonstrate that the usage of this benzoxazine-based tackifier does not affect the toughening effect of the composites, and meanwhile, endows the fiber preform with an excellent preforming effect.

## Figures and Tables

**Table 1:** Spring-back angles of the bifunctional CF fabrics treated at 80 °C/45min in vacuum.

BT5501A Content (g/m^2^)	Spring-Back Angle/°			
	10 min	1 h	4 h	16 h
0	43.7 ± 5.9	48.3 ± 3.5	51.4 ± 5.3	52.1 ± 4.3
1	34.5 ± 1.8	35 ± 0.3	36.8 ± 0.5	37.8 ± 1.7
2	31.8 ± 0.1	33.3 ± 0.5	33.9 ± 0.4	35.9 ± 0.6
3	27.8 ± 1.3	31.2 ± 1.1	32.2 ± 1.3	32.7 ± 1.3
4	0	0	0	0
6	0	0	0	0

**Table 2:** Compression after impact (CAI) strength of the composite laminates.

Properties	Data				Mean Value
Pit depth of CF/BMI (mm)	0.88	0.87	0.95	0.89	$0.90_{-0.03}^{+0.05}$
Pit depth of CF/PEK-C/BMI (mm)	0.53	0.69	0.59	0.63	$0.61_{-0.08}^{+0.08}$
Pit depth of CF/BT5501A/PEK-C/BMI (mm)	0.73	0.75	0.74	0.55	$0.69_{-0.14}^{+0.06}$
CAI strength of CF/BMI (MPa)	171.0	162.3	153.6	142.9	$157.5_{-14.6}^{+13.5}$
CAI strength of CF/PEK-C/BMI (MPa)	230.0	206.9	226.0	208.0	$217.7_{-10.8}^{+12.3}$
CAI strength of CF/BT5501A/PEK-C/BMI (MPa)	215.2	207.4	205.8	216.4	$211.2_{-5.4}^{+5.2}$

## Data Availability

Not applicable.
